# 4-Eth­oxy­phenyl 4-[(meth­oxy­carbon­yl)­oxy]benzoate

**DOI:** 10.1107/S1600536810044661

**Published:** 2010-11-06

**Authors:** H. T. Srinivasa, H. C. Devarajegowda, H. K. Arunkashi, T. G. Meenakshi

**Affiliations:** a Raman Research Institute, C. V. Raman Avenue, Sadashivanagar, Bangalore 560 080, Karnataka, India; b Department of Physics, Yuvaraja’s College (Constituent College), University of Mysore, Mysore 570 005, Karnataka, India; c Department of Physics, Y. Y. D. Govt. First Grade College, Belur 573 115 Hassan, Karnataka, India

## Abstract

In the title compound, C_17_H_16_O_6_, the two benzene rings form a dihedral angle of 54.95 (10)°. Only weak inter­molecular inter­actions are present in the crystal structure, *viz.* C—H⋯O hydrogen bonds and C—H⋯π inter­actions involving one of the benzene rings.

## Related literature

For general background to meth­oxy­carbon­yl(­oxy)benzoates, see Petrov (2002 ▶); Goodby *et al.* (1998 ▶); Castellano *et al.* (1971 ▶).
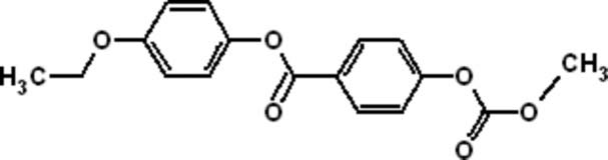


## Experimental

### 

#### Crystal data

C_17_H_16_O_6_
*M* *_r_* = 316.30
Monoclinic, 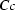

*a* = 11.7397 (5) Å
*b* = 16.9703 (6) Å
*c* = 7.9324 (3) Å
β = 96.949 (4)°
*V* = 1568.73 (11) Å^3^
*Z* = 4
Mo *K*α radiation
μ = 0.10 mm^−1^
*T* = 293 K
0.22 × 0.15 × 0.12 mm

#### Data collection

Oxford Diffraction Xcalibur diffractometer
Absorption correction: multi-scan (*CrysAlis PRO RED*; Oxford Diffraction, 2009 ▶) *T*_min_ = 0.982, *T*_max_ = 0.988
8839 measured reflections
1797 independent reflections
1092 reflections with *I* > 2σ(*I*)
*R*_int_ = 0.038

#### Refinement

*R*[*F* ^2^ > 2σ(*F* ^2^)] = 0.035
*wR*(*F* ^2^) = 0.085
*S* = 0.98
1797 reflections
211 parameters
2 restraints
H-atom parameters constrained
Δρ_max_ = 0.13 e Å^−3^
Δρ_min_ = −0.11 e Å^−3^

### 

Data collection: *CrysAlis PRO CCD* (Oxford Diffraction, 2009 ▶); cell refinement: *CrysAlis PRO CCD*; data reduction: *CrysAlis PRO RED* (Oxford Diffraction, 2009 ▶); program(s) used to solve structure: *SHELXS97* (Sheldrick, 2008 ▶); program(s) used to refine structure: *SHELXL97* (Sheldrick, 2008 ▶); molecular graphics: *ORTEP-3* (Farrugia, 1997 ▶) and *CAMERON* (Watkin *et al.*, 1993 ▶); software used to prepare material for publication: *WinGX* (Farrugia, 1999 ▶).

## Supplementary Material

Crystal structure: contains datablocks I, global. DOI: 10.1107/S1600536810044661/fb2212sup1.cif

Structure factors: contains datablocks I. DOI: 10.1107/S1600536810044661/fb2212Isup2.hkl

Additional supplementary materials:  crystallographic information; 3D view; checkCIF report

## Figures and Tables

**Table 1:** Hydrogen-bond geometry (Å, °) *Cg*1 is the centroid of the benzene ring C2,C4–C8.

*D*—H⋯*A*	*D*—H	H⋯*A*	*D*⋯*A*	*D*—H⋯*A*
C16—H16*B*⋯O2^i^	0.97	2.56	3.399 (4)	145
C1—H1*B*⋯*Cg*1^ii^	0.96	2.99	3.853 (4)	151

Symmetry codes: (i) 
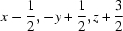
; (ii) 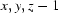.

**Fig. 1.**
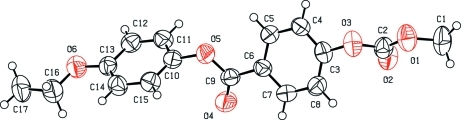
The title molecule with the displacement ellipsoids drawn at the 50% probability level. The H atoms are shown as spheres of arbitrary radii.

**Fig. 2.**
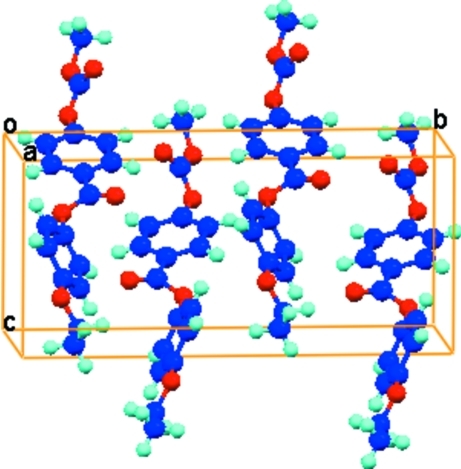
A view of the structure along the axis a.

## supplementary crystallographic information

### Comment

Liquid crystals play important role in technology. Among others, uniaxial calamitic (rod-like) nematic liquid crystals are active switching ingredients for the current LCD technology. A comparative study of physico-chemical and electro-optical properties of achiral calamitic liquid crystals which terminal, bridging and lateral alkoxy groups, with the corresponding alkyl group substituents has been carried out by Petrov (2002). It is well known that the terminal alkoxy substituent does not substantially affect the mesophase behaviour (Goodby *et al.*, 1998). Methyl and propyl group derivatives with respect to the title compound, *i. e.* 4-methoxyphenyl 4-[(methoxycarbonyl)oxy]benzoate and 4-propoxyphenyl 4-[(methoxycarbonyl)oxy]benzoate, respectively, do not form liquid-crystal phase. On the other hand, the title compound with the ethyl group forms a stable nematic phase (Castellano *et al.*, 1971). With this background, we have synthesized the title compound, 4-ethoxyphenyl 4-[(methoxycarbonyl)oxy]benzoate, and herein we report its crystal structure.

The asymmetric unit of the 4-ethoxyphenyl 4-[(methoxycarbonyl)oxy] benzoate, C_17_H_16_O_6_, contains just one molecule (Fig. 1). The two aromatic rings are non-coplanar; the interplanar angle between the two benzene rings (C3\C4\···\C8) and (C10\C11\···C15) equals to 54.95 (10)°. There are only weak intermolecular interactions in the structure: C—H···O hydrogen bonds (Tab. 1) as well as one C—H···π-ring electron ring interaction (Tab. 1). The packing of the molecules in the title structure is depicted in Fig. 2.

### Experimental

The 4-ethoxyphenyl 4-[(methoxycarbonyl)oxy]benzoate was synthesized according to the procedure described by Castellano *et al.* (1971). The crude white material was subjected to column chromatography using 60–120 mesh silica gel with ethyl acetate (1 ml) and hexane (99 ml) as an eluent. The retention factor equalled to 0.84. Single crystals, suitable for X-ray diffraction analysis, were obtained by recrystallization from pure hexane at room temperature. The yield was about 85%. M.p. 356 K.

Spectral data IR (KBr) cm^-1^: 2924 and 2852(CH_2_ aliphatic), 1759(OC═O ester), 1732(C═O ester), 1604 (aryl C═C), 1462(CH aryl). Elemental analysis: Theor.: C 64.55%, H 5.10%; Found: C 65.01%, H 4.72%.

### Refinement

All the H atoms were observable in the difference electron density maps except for just one H from each triplet of the methyl H atoms. Nevertheless, the H atoms were situated into the idealized positions and constrained by the riding atom approximation. The constraints: C_aryl_—H_aryl_ = 0.93, C_methylene_—H_methylene_ = 0.97 and C_methyl_—H_methyl_ = 0.96 Å. *U*_iso_(H_methyl_) = 1.5*U*_eq_(C_methyl_) or *U*_iso_(H_methylene_/_aryl_) = 1.2*U*_eq_(C_methylene_/_aryl_). In the absence of significant anomalous scattering effects, 1654 Friedel pairs were merged.

### Crystal data

**Table:**

C_17_H_16_O_6_	*F*(000) = 664
*M**_r_* = 316.30	*D*_x_ = 1.339 Mg m^−^^3^
Monoclinic, *C**c*	Melting point: 356 K
Hall symbol: C -2yc	Mo *K*α radiation, λ = 0.71073 Å
*a* = 11.7397 (5) Å	Cell parameters from 1797 reflections
*b* = 16.9703 (6) Å	θ = 2.4–27.5°
*c* = 7.9324 (3) Å	µ = 0.10 mm^−^^1^
β = 96.949 (4)°	*T* = 293 K
*V* = 1568.73 (11) Å^3^	Plate, colourless
*Z* = 4	0.22 × 0.15 × 0.12 mm

### Data collection

**Table:**

Oxford Diffraction Xcalibur diffractometer	1797 independent reflections
Radiation source: Enhance (Mo) X-ray Source	1092 reflections with *I* > 2σ(*I*)
graphite	*R*_int_ = 0.038
Detector resolution: 16.0839 pixels mm^-1^	θ_max_ = 27.5°, θ_min_ = 2.4°
ω scans	*h* = −14→15
Absorption correction: multi-scan (*CrysAlis PRO RED*; Oxford Diffraction, 2009)	*k* = −21→21
*T*_min_ = 0.982, *T*_max_ = 0.988	*l* = −10→10
8839 measured reflections	

### Refinement

**Table:**

Refinement on *F*^2^	Secondary atom site location: difference Fourier map
Least-squares matrix: full	Hydrogen site location: difference Fourier map
*R*[*F*^2^ > 2σ(*F*^2^)] = 0.035	H-atom parameters constrained
*wR*(*F*^2^) = 0.085	*w* = 1/[σ^2^(*F*_o_^2^) + (0.0418*P*)^2^] where *P* = (*F*_o_^2^ + 2*F*_c_^2^)/3
*S* = 0.98	(Δ/σ)_max_ < 0.001
1797 reflections	Δρ_max_ = 0.13 e Å^−^^3^
211 parameters	Δρ_min_ = −0.11 e Å^−^^3^
2 restraints	Extinction correction: *SHELXL97* (Sheldrick, 2008)
62 constraints	Extinction coefficient: 0.0052 (7)
Primary atom site location: structure-invariant direct methods	

### Special details

**Table:**

Experimental. *CrysAlisPro*, Oxford Diffraction (2010), Empirical absorption correction using spherical harmonics, implemented in SCALE3 ABSPACK scaling algorithm.
Geometry. All e.s.d.'s (except the e.s.d. in the dihedral angle between two l.s. planes) are estimated using the full covariance matrix. The cell e.s.d.'s are taken into account individually in the estimation of e.s.d.'s in distances, angles and torsion angles; correlations between e.s.d.'s in cell parameters are only used when they are defined by crystal symmetry. An approximate (isotropic) treatment of cell e.s.d.'s is used for estimating e.s.d.'s involving l.s. planes.
Refinement. Refinement of *F*^2^ against ALL reflections. The weighted *R*-factor *wR* and goodness of fit *S* are based on *F*^2^, conventional *R*- factors *R* are based on *F*, with *F* set to zero for negative *F*^2^. The threshold expression of *F*^2^ > σ(*F*^2^) is used only for calculating *R*-factors(gt) *etc*. and is not relevant to the choice of reflections for refinement. *R*-factors based on *F*^2^ are statistically about twice as large as those based on *F*, and *R*-factors based on ALL data will be even larger.

### Fractional atomic coordinates and isotropic or equivalent isotropic displacement parameters (Å^2^)

**Table:**

	*x*	*y*	*z*	*U*_iso_*/*U*_eq_	
O1	0.75904 (19)	0.40401 (15)	0.0153 (3)	0.0827 (7)	
O2	0.5908 (2)	0.34329 (14)	0.0242 (3)	0.0794 (7)	
O3	0.68466 (18)	0.40619 (14)	0.2481 (3)	0.0749 (6)	
O4	0.3150 (2)	0.27835 (12)	0.7091 (3)	0.0812 (7)	
O5	0.3405 (2)	0.40346 (11)	0.7901 (3)	0.0681 (6)	
O6	0.03480 (19)	0.39014 (13)	1.2645 (3)	0.0725 (6)	
C1	0.7605 (3)	0.3799 (3)	−0.1598 (5)	0.0961 (12)	
H1A	0.8087	0.4149	−0.2145	0.144*	
H1B	0.6839	0.3815	−0.2180	0.144*	
H1C	0.7898	0.3271	−0.1629	0.144*	
C2	0.6705 (3)	0.37896 (19)	0.0872 (4)	0.0630 (8)	
C3	0.5995 (3)	0.3876 (2)	0.3513 (3)	0.0604 (8)	
C4	0.5405 (3)	0.44911 (18)	0.4107 (4)	0.0610 (8)	
H4	0.5522	0.5002	0.3743	0.073*	
C5	0.4636 (2)	0.43453 (17)	0.5251 (4)	0.0587 (8)	
H5	0.4253	0.4763	0.5693	0.070*	
C6	0.4428 (2)	0.35787 (17)	0.5747 (3)	0.0523 (7)	
C7	0.5019 (3)	0.29643 (17)	0.5101 (4)	0.0610 (8)	
H7	0.4877	0.2449	0.5415	0.073*	
C8	0.5816 (3)	0.31082 (18)	0.3996 (4)	0.0653 (8)	
H8	0.6224	0.2696	0.3583	0.078*	
C9	0.3606 (2)	0.34004 (17)	0.6952 (4)	0.0549 (7)	
C10	0.2640 (3)	0.39527 (16)	0.9136 (4)	0.0577 (8)	
C11	0.1571 (3)	0.42874 (17)	0.8805 (4)	0.0639 (8)	
H11	0.1354	0.4534	0.7769	0.077*	
C12	0.0830 (3)	0.42581 (17)	0.9999 (4)	0.0620 (8)	
H12	0.0105	0.4482	0.9774	0.074*	
C13	0.1157 (3)	0.38941 (16)	1.1553 (4)	0.0530 (7)	
C14	0.2230 (3)	0.35663 (17)	1.1868 (4)	0.0635 (8)	
H14	0.2455	0.3323	1.2905	0.076*	
C15	0.2983 (3)	0.35972 (17)	1.0643 (4)	0.0628 (8)	
H15	0.3712	0.3377	1.0855	0.075*	
C16	0.0587 (3)	0.3503 (2)	1.4190 (5)	0.0816 (10)	
H16A	0.1238	0.3744	1.4868	0.098*	
H16B	0.0773	0.2957	1.3989	0.098*	
C17	−0.0464 (3)	0.3550 (2)	1.5115 (5)	0.0886 (12)	
H17A	−0.0310	0.3294	1.6198	0.133*	
H17B	−0.1097	0.3294	1.4454	0.133*	
H17C	−0.0654	0.4093	1.5281	0.133*	

### Atomic displacement parameters (Å^2^)

**Table:**

	*U*^11^	*U*^22^	*U*^33^	*U*^12^	*U*^13^	*U*^23^
O1	0.0645 (15)	0.1165 (18)	0.0703 (16)	−0.0045 (12)	0.0212 (12)	−0.0007 (14)
O2	0.0704 (15)	0.1012 (17)	0.0680 (14)	−0.0119 (13)	0.0132 (12)	−0.0162 (13)
O3	0.0674 (15)	0.0997 (16)	0.0590 (14)	−0.0168 (11)	0.0135 (11)	−0.0106 (12)
O4	0.1030 (17)	0.0546 (13)	0.0918 (16)	−0.0184 (11)	0.0355 (13)	−0.0090 (11)
O5	0.0850 (15)	0.0569 (12)	0.0676 (14)	−0.0092 (11)	0.0297 (12)	−0.0046 (11)
O6	0.0739 (15)	0.0778 (14)	0.0674 (15)	−0.0020 (11)	0.0156 (12)	0.0002 (12)
C1	0.085 (3)	0.138 (3)	0.071 (2)	0.009 (2)	0.033 (2)	−0.009 (2)
C2	0.053 (2)	0.075 (2)	0.062 (2)	0.0094 (17)	0.0106 (17)	−0.0008 (18)
C3	0.058 (2)	0.075 (2)	0.0478 (19)	−0.0021 (15)	0.0066 (15)	0.0024 (16)
C4	0.079 (2)	0.0527 (17)	0.0517 (17)	−0.0014 (15)	0.0077 (16)	0.0049 (14)
C5	0.0702 (19)	0.0516 (18)	0.0547 (19)	0.0049 (14)	0.0093 (16)	−0.0020 (14)
C6	0.0586 (18)	0.0494 (18)	0.0479 (18)	0.0016 (13)	0.0025 (15)	0.0032 (13)
C7	0.0687 (19)	0.0477 (17)	0.067 (2)	0.0072 (14)	0.0084 (16)	0.0037 (14)
C8	0.073 (2)	0.064 (2)	0.0593 (18)	0.0156 (15)	0.0096 (16)	−0.0022 (15)
C9	0.0625 (18)	0.0454 (17)	0.0558 (18)	0.0004 (14)	0.0033 (15)	−0.0005 (14)
C10	0.066 (2)	0.0492 (17)	0.061 (2)	−0.0088 (14)	0.0169 (16)	−0.0036 (14)
C11	0.079 (2)	0.0572 (17)	0.0544 (19)	0.0038 (16)	0.0036 (17)	0.0043 (13)
C12	0.0573 (18)	0.0614 (17)	0.067 (2)	0.0067 (14)	0.0057 (16)	−0.0013 (15)
C13	0.0556 (18)	0.0513 (17)	0.0528 (19)	−0.0075 (13)	0.0094 (15)	−0.0069 (14)
C14	0.071 (2)	0.068 (2)	0.0498 (18)	−0.0017 (16)	0.0004 (16)	0.0023 (15)
C15	0.0602 (18)	0.0637 (18)	0.064 (2)	0.0005 (14)	0.0072 (16)	0.0007 (16)
C16	0.093 (3)	0.087 (2)	0.066 (2)	−0.003 (2)	0.016 (2)	0.000 (2)
C17	0.090 (3)	0.110 (3)	0.071 (2)	−0.017 (2)	0.031 (2)	−0.003 (2)

### Geometric parameters (Å, °)

**Table:**

O1—C2	1.315 (4)	C6—C9	1.470 (4)
O1—C1	1.450 (4)	C7—C8	1.378 (4)
O2—C2	1.174 (4)	C7—H7	0.9300
O3—C2	1.349 (4)	C8—H8	0.9300
O3—C3	1.403 (3)	C10—C15	1.356 (4)
O4—C9	1.187 (3)	C10—C11	1.373 (4)
O5—C9	1.350 (3)	C11—C12	1.362 (4)
O5—C10	1.414 (3)	C11—H11	0.9300
O6—C13	1.361 (3)	C12—C13	1.390 (4)
O6—C16	1.398 (4)	C12—H12	0.9300
C1—H1A	0.9600	C13—C14	1.373 (4)
C1—H1B	0.9600	C14—C15	1.391 (4)
C1—H1C	0.9600	C14—H14	0.9300
C3—C4	1.368 (4)	C15—H15	0.9300
C3—C8	1.381 (4)	C16—C17	1.512 (5)
C4—C5	1.378 (4)	C16—H16A	0.9700
C4—H4	0.9300	C16—H16B	0.9700
C5—C6	1.389 (4)	C17—H17A	0.9600
C5—H5	0.9300	C17—H17B	0.9600
C6—C7	1.385 (4)	C17—H17C	0.9600
			
C2—O1—C1	115.2 (3)	O4—C9—C6	125.5 (3)
C2—O3—C3	117.4 (2)	O5—C9—C6	111.8 (2)
C9—O5—C10	118.4 (2)	C15—C10—C11	121.3 (3)
C13—O6—C16	118.2 (3)	C15—C10—O5	120.6 (3)
O1—C1—H1A	109.5	C11—C10—O5	118.0 (3)
O1—C1—H1B	109.5	C12—C11—C10	119.8 (3)
H1A—C1—H1B	109.5	C12—C11—H11	120.1
O1—C1—H1C	109.5	C10—C11—H11	120.1
H1A—C1—H1C	109.5	C11—C12—C13	120.1 (3)
H1B—C1—H1C	109.5	C11—C12—H12	119.9
O2—C2—O1	127.8 (3)	C13—C12—H12	119.9
O2—C2—O3	125.4 (3)	O6—C13—C14	125.7 (3)
O1—C2—O3	106.7 (3)	O6—C13—C12	114.9 (3)
C4—C3—C8	121.6 (3)	C14—C13—C12	119.4 (3)
C4—C3—O3	117.1 (3)	C13—C14—C15	120.3 (3)
C8—C3—O3	121.2 (3)	C13—C14—H14	119.9
C3—C4—C5	119.3 (3)	C15—C14—H14	119.9
C3—C4—H4	120.4	C10—C15—C14	119.1 (3)
C5—C4—H4	120.4	C10—C15—H15	120.5
C4—C5—C6	120.4 (3)	C14—C15—H15	120.5
C4—C5—H5	119.8	O6—C16—C17	108.1 (3)
C6—C5—H5	119.8	O6—C16—H16A	110.1
C7—C6—C5	119.2 (3)	C17—C16—H16A	110.1
C7—C6—C9	118.9 (3)	O6—C16—H16B	110.1
C5—C6—C9	121.9 (2)	C17—C16—H16B	110.1
C8—C7—C6	120.7 (3)	H16A—C16—H16B	108.4
C8—C7—H7	119.6	C16—C17—H17A	109.5
C6—C7—H7	119.6	C16—C17—H17B	109.5
C7—C8—C3	118.8 (3)	H17A—C17—H17B	109.5
C7—C8—H8	120.6	C16—C17—H17C	109.5
C3—C8—H8	120.6	H17A—C17—H17C	109.5
O4—C9—O5	122.7 (3)	H17B—C17—H17C	109.5
			
C1—O1—C2—O2	−4.3 (5)	C5—C6—C9—O4	158.2 (3)
C1—O1—C2—O3	179.3 (3)	C7—C6—C9—O5	158.5 (3)
C3—O3—C2—O2	2.9 (5)	C5—C6—C9—O5	−20.9 (3)
C3—O3—C2—O1	179.4 (3)	C9—O5—C10—C15	77.9 (3)
C2—O3—C3—C4	−117.8 (3)	C9—O5—C10—C11	−106.2 (3)
C2—O3—C3—C8	66.3 (4)	C15—C10—C11—C12	−0.7 (4)
C8—C3—C4—C5	1.8 (4)	O5—C10—C11—C12	−176.5 (2)
O3—C3—C4—C5	−174.0 (3)	C10—C11—C12—C13	0.4 (4)
C3—C4—C5—C6	−2.4 (4)	C16—O6—C13—C14	−4.1 (4)
C4—C5—C6—C7	1.1 (4)	C16—O6—C13—C12	176.0 (3)
C4—C5—C6—C9	−179.5 (3)	C11—C12—C13—O6	179.9 (2)
C5—C6—C7—C8	0.8 (4)	C11—C12—C13—C14	0.0 (4)
C9—C6—C7—C8	−178.6 (3)	O6—C13—C14—C15	−180.0 (2)
C6—C7—C8—C3	−1.5 (5)	C12—C13—C14—C15	−0.1 (4)
C4—C3—C8—C7	0.1 (4)	C11—C10—C15—C14	0.6 (4)
O3—C3—C8—C7	175.8 (3)	O5—C10—C15—C14	176.3 (2)
C10—O5—C9—O4	1.5 (4)	C13—C14—C15—C10	−0.2 (4)
C10—O5—C9—C6	−179.3 (2)	C13—O6—C16—C17	−176.2 (3)
C7—C6—C9—O4	−22.4 (4)		

### Hydrogen-bond geometry (Å, °)

**Table:**

Cg1 is the centroid of the benzene ring C2,C4–C8.

**Table:**

*D*—H···*A*	*D*—H	H···*A*	*D*···*A*	*D*—H···*A*
C16—H16B···O2^i^	0.97	2.56	3.399 (4)	145
C1—H1B···Cg1^ii^	0.96	2.99	3.853 (4)	151

Symmetry codes: (i) *x*−1/2, −*y*+1/2, *z*+3/2; (ii) *x*, *y*, *z*−1.
